# Accumulation and fractionation of rare earth elements are conserved traits in the *Phytolacca* genus

**DOI:** 10.1038/s41598-019-54238-3

**Published:** 2019-12-05

**Authors:** Nicolas Grosjean, Marie Le Jean, Charlotte Berthelot, Michel Chalot, Elisabeth Maria Gross, Damien Blaudez

**Affiliations:** 1 Université de Lorraine, CNRS, LIEC, F-54000, Nancy, France; 2 Université de Lorraine, CNRS, LIEC, F-57000, Metz, France; 3 CTIFL, ZI Belle Etoile, F-44483, Carquefou, France; 4 Université de Bourgogne Franche-Comté, UMR CNRS 6249 Laboratoire Chrono-environnement, F-25211, Montbéliard, France; 5 Université de Lorraine, F-54000, Nancy, France

**Keywords:** Plant physiology, Abiotic

## Abstract

Rare earth elements (REEs) are now considered emerging pollutants in the environment. *Phytolacca americana*, an REE hyperaccumulating plant, has been proposed for the remediation of REE-contaminated soils. However, there is no REE-related information for other *Phytolacca* species. Here, we examined five species (*P. americana*, *P. acinosa*, *P. clavigera*, *P. bogotensis*, and *P. icosandra*) for their response to REEs. REE accumulation and fractionation traits both occurred on the same order of magnitude among the five species. Heavy REEs were preferentially transferred to leaves relative to light REEs. Regardless of the species, lateral root length and chlorophyll content decreased under REE exposure, and lateral roots and foliar anthocyanins increased. However, plants did not experience or only slightly experienced oxidative stress. Finally, REE exposure strongly modulated the ionome of roots and, to a lesser extent, that of leaves, with a negative correlation between REE and Mn contents. In conclusion, our study provides new data on the response of several *Phytolacca* species to REEs. Moreover, we highlighted that the REE accumulation trait was conserved among *Phytolacca* species. Thus, we provide valuable information for the phytoremediation of REE-contaminated sites since the most appropriate *Phytolacca* species could be selected depending on the climatic/pedological area to be remediated.

## Introduction

The Rare Earth Elements (REEs) are a group of 17 metallic elements including the 15 lanthanides plus yttrium and scandium. REEs can be further split into two groups known as the light REEs (LREEs, spanning from lanthanum (La) to europium (Eu), plus scandium (Sc)) and the heavy REEs (HREEs, spanning from gadolinium (Gd) to lutetium (Lu), plus yttrium (Y)). REEs are widely found in the Earth’s crust. Cerium (Ce), La, neodymium (Nd) and Y are the most abundant REEs and can be found at concentrations similar to those of Zn, Cu, Ni and Mo, among others^1^. A fast increase in the demand and subsequent production of REEs has been observed over the past decades because of their diverse use in new technologies, green energies and medical devices^2^. Moreover, the very low recyclability of these elements^3^, their use as food additives for livestock and poultry^4^, their spreading as fertilizers in agriculture^1^ or as eutrophication regulators^5^, along with the high soil concentrations found in mining areas^6,7^ lead REEs to be considered emerging pollutants.

Although these elements are not essential to plants, REEs can be detected at moderate concentrations under non-contaminated conditions in plant tissues. The REE concentrations in leaves ranged from 0.0011 (Lu) to 0.33 mg/kg DW (Ce)^8^ and even up to 5 mg REE/kg DW^9^. However, as reported for other metals, several plant species, mainly ferns, are able to accumulate REEs. Two fern species displayed the highest REE accumulation potential. *Dicranopteris dichotoma* (syn. *D. linearis*) accumulated up to 0.7% DW of LREEs^6,10^ and *Pronephrium simplex* accumulated 1.2 g REE/kg DW^11,12^. To a lesser extent, other ferns could also accumulate REEs, such as *Dryopteris erythrosora*^9^, *Blechnum orientale*^13,14^ and *Athyrium yokoscense*^15^. Although ferns are highly represented, a few angiosperms are also known to accumulate REEs. *Carya tomentosa* (mockernut) accumulated REEs up to 859 mg/kg in a non-contaminated environment^14,16^. *Phytolacca americana* (pokeweed), first identified as a Mn hyperaccumulator^17–19^, with a Mn accumulation up to 2000 mg REE/kg DW on non-contaminated soils^17^, was further reported as an REE accumulator^15,20^.

In comparison to other REE-accumulating species, *P. americana* is a fast growing and high-biomass producing plant that can reach 3 m in height. This ubiquitous weed of roadsides and disturbed areas in its native range of the southeastern United States is now distributed worldwide^17^. Notably, this species was found at an REE mining site in southern Jiangxi Province in China, with an average REE concentration of approximately 250 mg/kg in leaves, reaching up to 1,040 mg/kg^7^. Recently, several studies investigated the translocation and fractionation of REEs in *P. americana*. Yuan *et al*. (2017, 2018) observed a higher translocation of HREEs in the leaves compared to LREEs, while more LREEs accumulated in the roots and stems. Organic or amino acids have been implicated in complexing REEs and participating in the long-distance transport of these elements in *P. americana*^20,21^. Combined, these features would be of great interest for the phytoremediation of REE-contaminated soils. In addition, REEs extracted from plants could be further purified^22^ or used for ecocatalysis^23^.

Combining ecological and genetic analyses of *P. americana*, no genetic differentiation could be detected between populations from Mn-contaminated and uncontaminated sites^24^, suggesting that phenotypic plasticity is probably the major contributor to the successful colonization of marginal lands, such as metal-contaminated soils. The related species *P. acinosa*^25,26^ is also a Mn hyperaccumulator, showing that the Mn hyperaccumulation trait is found in different species of *Phytolacca*. Similar findings were reported concerning the Ni hyperaccumulation trait in several species of *Alyssum* and *Cochlearia*^27^. Likewise, several *Carya* species accumulated similar REE concentrations in leaves^16^, therefore highlighting that the REE accumulation trait could be conserved throughout the *Carya* genus. However, the conservation of this REE accumulation potential has not yet been investigated for *Phytolacca* species other than *P. americana*.

Consequently, we propose the hypothesis of a monophyletic REE accumulation trait within the *Phytolacca* genus. Therefore, we analysed the REE accumulation potential as well as the REE fractionation pattern of five *Phytolacca* species (*P. americana, P. acinosa, P. bogotensis, P. clavigera* and *P. icosandra*). Our analyses also aimed to reveal how REE accumulation in these species of *Phytolacca* modified the elemental composition of roots and shoots. In addition, analyses were performed to study the effects of REEs on a set of biomarkers related to modification of root architecture, leaf pigment composition and oxidative stress.

## Results and Discussion

### Molecular confirmation of the *Phytolacca* species

**Figure 1:**
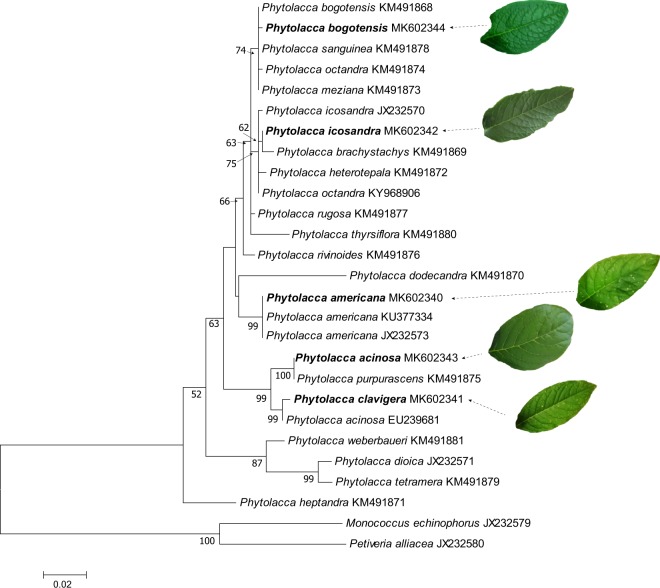
Maximum likelihood phylogram for the identification of *Phytolacca* species used based on ITS1-5.8S-ITS2 sequences. Aligned sequences using ClustalW were used to build a maximum likelihood tree based on the Kimura 2-parameter method. Bootstrap values over 50% (1000 replicates) are indicated below the branches. Analyses were conducted in MEGA7^54^. The ITS sequences of the *Phytolacca* species used in this study are indicated in bold and compared with other *Phytolacca* species available in GenBank. *Monococcus echinophorus* and *Petiveria alliacea* were used as outgroups. The leaf morphology of the tested species is shown.

Seeds from five species of *Phytolacca* were obtained from commercial or institutional origins as described in the materials and methods section and identified as *P. americana*, *P. acinosa, P. bogotensis*, *P. clavigera*, and *P. icosandra*. The identification of these species was based on morphological traits. However, due to the ambiguous taxonomy within the *Phytolacca* genus^28^, we performed a molecular investigation to confirm that the species tested were different. For this purpose, the ITS region was amplified, sequenced and compared with ITS sequences of *Phytolacca* species retrieved from GenBank. For most species, there was a single ITS sequence available in the database, and none were available for *P. clavigera* (Fig. 1). For *P. bogotensis*, *P. icosandra* and *P. americana*, our data matched the previously published ITS sequences for these species. However, it was less straightforward for *P. acinosa* and *P. clavigera*, where a mis-identification might have been made. Facing this problem, we analysed a second seed batch of *P. acinosa* obtained from a different supplier. Both plant morphology and ITS sequences were identical for the two batches, suggesting a correct identification of *P. acinosa*. Notably, different leaf morphologies were observed between *P. acinosa* and *P. clavigera* (Fig. 1), which shared the highest identity (97%). Leaves of *P. acinosa* were obovate, whereas the four other species had oval-shaped leaves. In conclusion, the molecular analyses confirmed that the five species tested were different. Sequence data were deposited in GenBank under the following accession numbers: MK602340 (*P. americana*), MK602343 (*P. acinosa*), MK602344 (*P. bogotensis*), MK602341 (*P. clavigera*), and MK602342 (*P. icosandra*).

### The REE accumulation trait was shared among *Phytolacca* species

**Figure 2:**
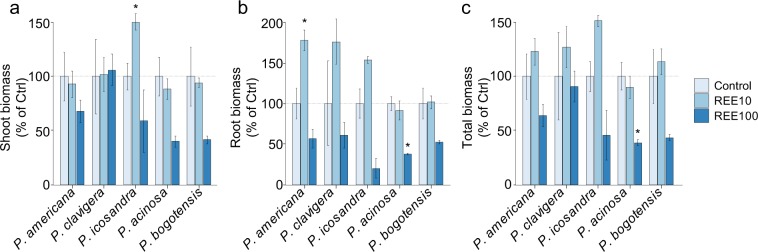
Effect of REEs on the biomass of *Phytolacca* species. Biomass (DW) is expressed as a percentage of that of the control (no REE) in shoots (**a**), roots (**b**) and total plants (**c**) exposed to 10 µM or 100 µM REEs (REE10 and REE100, respectively). The data are the means (±SE) of n = 3 (control, REE10) or n = 4 (REE100) plants. Significant differences from the control condition are indicated by asterisks (P < 0.05, t-test).

We first investigated the potential of the different *Phytolacca* species to accumulate REEs in their tissues. To this end, *Phytolacca* seedlings were grown hydroponically and exposed to a mixture of 16 REEs supplied at equimolar concentrations. Diatloff *et al*. (1996) reported that in uncontaminated sites, REE concentrations from the soil solution can reach the micromolar range^29^. Colim *et al*. (2019) analyzed the REE concentrations in surface water samples from the Lavras do Sul (Brazil) mining region. The total REE concentrations measured ranged from 27 to 279 µM^30^. Therefore, we decided to use two different REE concentrations, a low and a high concentration (10 and 100 µM). These concentrations were also pre-determined from preliminary experiments (data not shown), for which biomass was either not affected or reduced. Indeed, at the concentration of 10 µM REEs in the nutrient solution (REE10), no negative effect was observed for root, shoot and total plant biomass for any species (Fig. 2). A significant positive effect on the shoot biomass of *P. icosandra* and the root biomass of *P. americana* was observed even at this lowest concentration. Conversely, at 100 µM REEs (REE100), biomass tended to decrease by approximately 50% for most species, despite not always being significant (Fig. 2). These results suggest a similar REE tolerance level of the five *Phytolacca* species.

**Figure 3:**
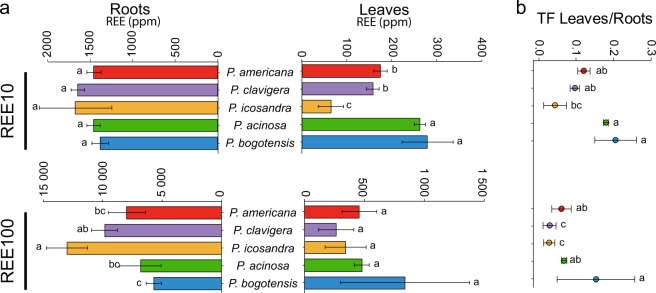
REE accumulation by five *Phytolacca* species. Plants were exposed to a mixture of 10 µM or 100 µM REEs (REE10 and REE100, respectively). (**a**) REE concentrations in roots and leaves of *Phytolacca* species. (**b**) Translocation factor (TF) of REEs from the roots to the leaves. The data are the means (±SD) of n = 3 (REE10) or n = 4 (REE100). Within a given treatment, values with the same letter are not significantly different (P < 0.05, ANOVA, Tukey’s HSD).

Then, REE accumulation was determined by ICP-MS, and the results are given in Fig. 3. At REE10, the five species accumulated approximately 1500 mg/kg REEs in the roots, and no significant difference was observed between the species (Fig. 3a). However, at REE100, an overall higher accumulation was found, and differences between species could be noted (Fig. 3a). *Phytolacca icosandra* displayed the highest concentration of approximately 13,000 mg/kg REEs, while *P. acinosa* and *P. bogotensis* accumulated 2.4 and 2.3 times less REEs, respectively (Fig. 3a). *P. clavigera* and *P. americana* had intermediate concentrations in their roots.

In leaves, the patterns of REE accumulation differed from those of roots. At REE10, *P. bogotensis* and *P. acinosa* were the two species accumulating the highest concentrations of REEs, with 279 and 261 mg/kg, respectively. *Phytolacca americana* and *Phytolacca clavigera* accumulated slightly less, with 174 and 157 mg/kg, respectively, while the lowest concentration was recorded for *P. icosandra*, with less than 100 mg/kg (Fig. 3a). However, those variations were not observed at REE100. Despite a mean accumulation value ranging from 265 mg/kg (*P. clavigera*) to 837 mg/kg (*P. bogotensis*), these values were not significantly different (Fig. 3a). Our results with *P. americana* are consistent with those from a previous study, carried out with the same species also grown hydroponically, where a similar REE accumulation capacity was reported^21^. As a comparison, *P. americana* accumulated in the leaves at approximately 300 and 500 mg/kg REEs when exposed to 10 and 100 µM REEs, respectively^21^.

For each species, the translocation factor (TF) of REEs from roots to leaves was further calculated (Fig. 3b). Under both REE concentrations, *P. acinosa* and *P. bogotensis* had the highest TFs, whereas *P. clavigera* and *P. icosandra* had the lowest TFs. However, the TF values were in the same range, and relatively low differences were found between the species (Fig. 3b). Moreover, our data are in agreement with those described by Yuan *et al*. (2017) for *P. americana*, where a TF of approximately 0.1 was reported^21^.

Overall, our results suggest that the accumulation of REEs in the leaves and their translocation from roots to shoots are of the same order of magnitude in the five *Phytolacca* species.

### HREEs were preferentially translocated to leaves in the five *Phytolacca* species

**Figure 4:**
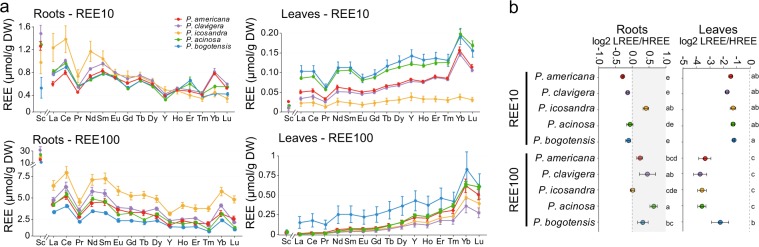
REE fractionation in roots and leaves of *Phytolacca* species. Plants were exposed to a mixture of 10 µM or 100 µM REEs (REE10 and REE100, respectively). (**a**) REE concentration patterns in roots and leaves of *Phytolacca* species (the linkage of data points does not indicate dependence). (**b**) Concentration ratios of LREEs (Sc, La to Eu) over HREEs (Gd to Lu, Y) among the species tested and exposed either to 10 µM or 100 µM as indicated. The data are the means (±SD) of n = 3 (REE10) or n = 4 (REE100). Within a given treatment, values with the same letter are not significantly different (P < 0.05, ANOVA, Tukey’s HSD).

We further investigated whether various REE fractionation patterns (LREEs *vs* HREEs) could be mediated by the different *Phytolacca* species. Therefore, we analysed the fractionation pattern of the whole set of REEs in both roots and shoots of the five species. To compare the fractionation process for various REE species mediated by different plant species, we used our hydroponic system to ensure equimolar concentrations of all REE species in the nutritive medium. The data of REE fractionation are given in Fig. 4a. REEs are ordered by their decreasing ionic radii at their trivalent oxidation state. The non-lanthanide Y, generally included in the HREEs, thus ranged between Dy and Ho. Interestingly, the five *Phytolacca* species displayed very similar REE accumulation patterns. In roots, the REE concentration decreased from La to Lu (Fig. 4a). Conversely, in leaves, the REE concentration increased from La to Lu (Fig. 4a). These conclusions were true regardless of the *Phytolacca* species and the REE concentration (REE10, REE100). The only exceptions were praseodynium (Pr) and ytterbium (Yb), for which anomalies could be demonstrated. However, these anomalies were similar among the five *Phytolacca* species.

We further analysed the accumulation ratios between LREEs and HREEs (Fig. 4b). At REE10, there was no particular enrichment of LREEs *versus* HREEs in roots. At REE100, and except for *P. icosandra*, a slight LREE enrichment in roots was found for the different *Phytolacca* species. Conversely, leaves were highly enriched with HREEs regardless of the *Phytolacca* species considered. At REE10, HREE concentration was indeed between 2 and 4 times higher than that of LREEs. This enrichment was more pronounced at REE100, where HREEs were from 5 (*P. bogotensis*) to 13 (*P. clavigera*) times more abundant than LREEs (Fig. 4b). Altogether, the data indicated a preferential root-to-leaf translocation of HREEs *vs* LREEs, a trait that was conserved in the five *Phytolacca* species.

The preferential translocation of HREEs to shoots had already been observed for *P. americana* grown either under hydroponic conditions or naturally in REE-mining areas^7,21^. In non-accumulating species, a higher transfer of HREEs *vs* LREEs has also been reported in several angiosperms, notably wheat^31,32^, soybean^33^ and rice^34^. Conversely, LREE enrichment in shoots has been reported in fern species accumulating REEs. Likewise, non-accumulating ferns also had a higher content of LREEs than HREEs (Grosjean N., personal communication). Therefore, the different fractionation patterns described above are very unlikely to be related to the REE accumulation trait. More likely, angiosperms and pteridophytes, which are evolutionarily very distant, might not share the same mechanisms underlying REE translocation from roots to shoots. Such different fractionation processes could be explained by the production of specific compounds with distinct REE-chelating properties. This hypothesis was supported by previous works that reported that the REE-accumulating fern *D. dichotoma* produces a specific LREE-binding peptide^35^ and that HREE enrichment in the shoots of *P. americana* was associated with the long-distance transport of HREE-organic acid complexes, such as HREE-citrate^21^. However, to test this hypothesis, further studies investigating the ligands associated with REEs in the xylem sap of a panel of angiosperms and pteridophytes are needed.

### REEs impacted root architecture

**Figure 5:**
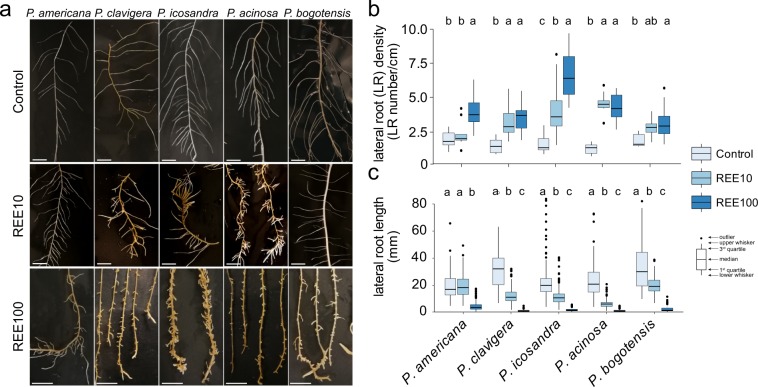
Effect of REEs on the root architecture of *Phytolacca* species. (**a**) Architecture of representative roots (scale bars = 1 cm), (**b**) lateral root branching (expressed as the number of lateral roots per cm of primary root) and (**c**) lateral root length of the different *Phytolacca* species exposed to a mixture of 10 µM or 100 µM REEs (REE10 and REE100, respectively) or not exposed to REEs (control). Root systems of three (control, REE10) or four (REE100) plants per species and per treatment were analysed. Within a given species, significant differences between treatments are indicated by different letters (P < 0.05, Kruskal-Wallis).

It has already been reported that high concentrations of La can inhibit root elongation and induce lateral root development in *Arabidopsis thaliana*^36^. This mechanism was explained by the accumulation of reactive oxygen species (ROS) in the root tip, leading to the death of cells from the primary root tips and reorientation of auxin to lateral roots^36,37^. Thus, to verify whether root elongation and branching were also affected by a mixture of REEs, the root architecture of the five *Phytolacca* species was analysed after exposure to 10 and 100 µM REEs and compared to control plants (Fig. 5). The overall root architecture of the different species was relatively similar under the control condition (Fig. 5a). The addition of REEs triggered modifications in the root architecture that were characterized by shorter lateral roots compared to the control condition (Fig. 5a). At REE10, morphological differences were noticed for *P. clavigera*, *P. icosandra*, and *P. acinosa* when compared to non-exposed plants. These differences were much less obvious in *P. bogotensis* and *P. americana*. However, at REE100, the root architecture of all species was more impacted, even if *P. americana* was the less affected species (Fig. 5a). The quantification of both the density and length of lateral roots confirmed the visual observations (Fig. 5b,c). The number of lateral roots indeed increased under REE exposure. However, for both *P. americana* and *P. bogotensis*, the values were only significantly different when comparing the control and REE100 conditions (Fig. 5b). Similarly, with increasing REE concentrations, the lateral root length decreased, with the exception of *P. americana*, which was not affected at REE10 when compared to the control (Fig. 5c). In conclusion, analysis of the root architecture of the different species allowed us to show that, despite a similar REE accumulation rate for the species tested, *P. americana* was the least affected by REEs.

### *Phytolacca* species did not experience or only slightly experienced oxidative stress under REE exposure

We further selected two markers related to oxidative stress, namely, the malondialdehyde (MDA) concentration and the total antioxidant capacity (TAC), to investigate the effect of REE exposure in the five species (Table 1).

**Table 1:** Total antioxidant capacity (TAC) and malondialdehyde (MDA) contents in leaves and roots of plants exposed to REEs.

		Treatment	*P. americana*	*P. clavigera*	*P. icosandra*	*P. acinosa*	*P. bogotensis*
**Leaves**	**TAC** (nmol/mg FW)	Control	5.06 ± 0.43^a^	4.40 ± 0.67^b^	4.66 ± 1.42^a^	3.50 ± 1.98^a^	2.92 ± 0.08^a^
		REE - 10	6.82 ± 1.23^a^	5.53 ± 0.09^a^	6.94 ± 0.63^a^	3.73 ± 0.72^a^	2.65 ± 0.13^a^
		REE - 100	6.05 ± 0.69^a^	3.35 ± 0.30^c^	6.53 ± 1.63^a^	4.04 ± 1.10^a^	2.62 ± 0.60^a^
	**MDA** (nmol/mg FW)	Control	1.04 ± 0.06^a^	0.81 ± 0.05^a^	0.66 ± 0.18^b^	0.98 ± 0.13^a^	1.16 ± 0.14^a^
		REE - 10	1.09 ± 0.18^a^	1.35 ± 0.71^a^	1.08 ± 0.16^a^	0.42 ± 0.05^b^	0.47 ± 0.12^b^
		REE - 100	0.86 ± 0.15^a^	0.85 ± 0.07^a^	0.94 ± 0.09^ab^	0.84 ± 0.16^a^	1.20 ± 0.12^a^
		**Treatment**	**P. americana**	**P. clavigera**	**P. icosandra**	**P. acinosa**	**P. bogotensis**
**Roots**	**TAC** (nmol/mg FW)	Control	0.55 ± 0.07^a^	0.60 ± 0.12^ab^	0.47 ± 0.03^a^	0.64 ± 0.06^b^	0.48 ± 0.30^a^
		REE - 10	0.63 ± 0.20^a^	0.70 ± 0.15^a^	0.46 ± 0.07^a^	1.00 ± 0.05^a^	0.41 ± 0.05^a^
		REE - 100	0.99 ± 0.49^a^	0.38 ± 0.05^b^	0.33 ± 0.29^a^	0.70 ± 0.11^b^	0.62 ± 0.20^a^
	**MDA** (nmol/mg FW)	Control	0.16 ± 0.02^a^	0.20 ± 0.09^a^	0.09 ± 0.02^a^	0.16 ± 0.03^a^	0.17 ± 0.01^a^
		REE - 10	0.30 ± 0.20^a^	0.34 ± 0.13^a^	0.11 ± 0.02^a^	0.15 ± 0.05^a^	0.09 ± 0.03^a^
		REE - 100	0.18±0.04^a^	0.19 ± 0.02^a^	0.14 ± 0.02^a^	0.23 ± 0.10^a^	0.22 ± 0.05^a^

*Phytolacca* species were exposed to a mixture of 10 µM (REE10) (n = 3) or 100 µM REEs (REE100) (n = 4). Values are means ± SD. Within a given species, significant differences between treatments are indicated by different letters (P < 0.05, ANOVA Tukey’s HSD).

TAC represents the non-enzymatic antioxidant capacity and is indicative of the ability to counteract oxidative stress-induced damage in cells. In both roots and leaves of the different species, only a few differences were found among the different treatments (Table 1). Indeed, REE exposure only impacted the TAC (1.3-fold compared to the control) in the leaves of *P. clavigera*. Similarly, in the roots of *P. acinosa*, the TAC content increased by 1.6-fold at REE10 when compared to the control (Table 1).

The quantification of MDA, one of the end products of the chain reaction of lipid peroxidation, allows us to estimate potential oxidative damage. No significant change was observed in roots under REE exposure, regardless of the species considered. In the leaves of *P. acinosa* and *P. bogotensis*, the MDA concentration decreased by 2.5-fold at REE10 and to a lesser extent (1.3-fold) in *P. acinosa* at REE100. Conversely, it slightly increased (1.6-fold) in the leaves of *P. icosandra* at REE10 (Table 1).

Combined, MDA and TAC analyses suggest that *Phytolacca* species were not affected or were only slightly affected by oxidative stress when exposed to REEs. Conversely, several studies have demonstrated the generation of REE-induced oxidative stress in plant species that are not REE accumulators (e.g., *Nymphoides peltata* and *Hydrilla verticillata*), with, for example, a progressive increase in the concentration of MDA with increasing REE concentrations^38–40^. Therefore, our data suggest that the five *Phytolacca* species are most likely highly tolerant to REEs. However, additional studies will be needed to compare the REE tolerance levels of different *Phytolacca* species.

### REEs impacted the pigment contents and nitrogen balance index

**Figure 6:**
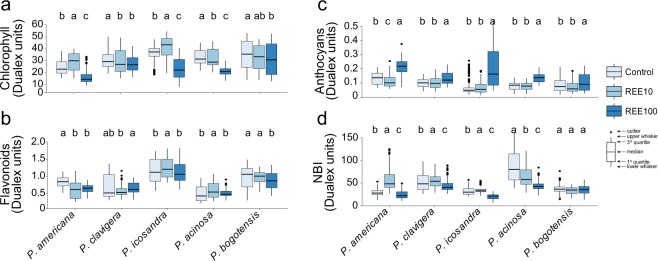
Pigment contents and nitrogen balance index of *Phytolacca* species exposed to REEs. (**a**) Chlorophyll, (**b**) flavonoid, (**c**) anthocyanin, and (**d**) nitrogen balance (NBI) indexes were measured in the leaves of *Phytolacca* species exposed to a mixture of 10 µM or 100 µM REEs (REE10 and REE100, respectively). Twenty measurements of three (control, REE10) or four (REE100) plants per species and per treatment were performed. Within a given species, significant differences between treatments are indicated by different letters (P < 0.05, Kruskal-Wallis, Wilcoxon post hoc test).

Given the high REE accumulation in leaves, we investigated its impact on the content of different pigments (chlorophyll, flavonoids, and anthocyanins) and on the nitrogen balance index (NBI). Differences between the treatments but also between the species were recorded (Fig. 6). Regardless of the species, the chlorophyll index moderately decreased at REE100 when compared to the control. Indeed, the chlorophyll content was reduced from 1.1 to 1.6 times in *P. bogotensis* and *P. icosandra*, respectively (Fig. 6a). The lower chlorophyll content under REE exposure could either be due to a reduction of Mg supply^40^ or to chlorophyll degradation caused by lipid peroxidation^39^. Since the MDA concentration did not increase at REE100 in the different *Phytolacca* species, this second hypothesis is very unlikely. However, it is noteworthy that the chlorophyll content increased for *P. icosandra* and *P. americana* at REE10 (Fig. 6a). Such a result could suggest a stimulation of photosynthesis. Several studies reported growth stimulation in rice^41^, tobacco^42^ and soybean^43^ at low REE concentrations.

Compared to chlorophylls, the flavonoid content was poorly affected by REE exposure (Fig. 6b). When compared to the control, it slightly increased at REE10 by 1.1 and 1.2 times in *P. icosandra* and *P. acinosa*, respectively. Conversely, it decreased in *P. americana* and *P. bogotensis* at either REE10 or REE100. The anthocyanin index, usually indicative of plant stress^44^, was also analysed and is reported in Fig. 6c. A similar pattern was found for the different species, where all species showed a significant increase in anthocyanins at REE100 when compared to the control (Fig. 6c). These data suggest that this high dose of REEs, associated with a subsequent high REE accumulation in leaves (Fig. 3a), could lead to a modification of anthocyanin biosynthesis. In addition, the increase in anthocyanin content could be caused by an increased limitation of phosphate in the plant due to the high reactivity of REEs with phosphates. Surprisingly, at REE10, *P. americana* was the only species for which there was a decrease of anthocyanins (Fig. 6c), suggesting that at this low REE exposure, the leaf stress was less important to *P. americana* than to its close relatives. The different pigment indexes measured can provide indications of the health or stress status of plants under tested conditions. It has been demonstrated in *Arabidopsis thaliana* that an increase of anthocyanins combined with a decrease of the chlorophyll content was triggered in response to metallic stress^45^. Indeed, anthocyanins can play a major protective role against metal stresses by acting as antioxidants^45^. However, since no difference was obtained from the MDA and TCA measurements, strong oxidative stress is unlikely, and a higher anthocyanin index of all five species measured at the highest REE concentration might reflect phosphorus deficiency, as suggested above and as previously reported^46^.

We also analysed the NBI in plants exposed to REEs. NBI is a marker that corresponds to the ratio of chlorophyll content to that of flavonoids and gives an indication of the nitrogen status of plants^47^. While no difference was noticed for *P. bogotensis*, the NBI slightly increased at REE10 for *P. americana*, *P. clavigera*, and *P. icosandra* (Fig. 6d). Except for *P. bogotensis*, the NBI decreased at REE100 in the four other species.

The relatively lower root branching, the increase of the chlorophyll content, along with the increased NBI for *P. americana* at REE10, could suggest that *P. americana* is slightly more tolerant to low REE exposure compared to the other species tested (Figs. 5, 6). However, since the growth of other *Phytolacca* species was not more affected by REEs than that of *P. americana* for both REE10 and REE100 (Fig. 2), REE tolerance levels of the different species should be similar. Further dose-response studies would, however, be needed to shed more light on the tolerance levels of the different *Phytolacca* species.

### REEs modulate the ionome of *Phytolacca* species

**Figure 7:**
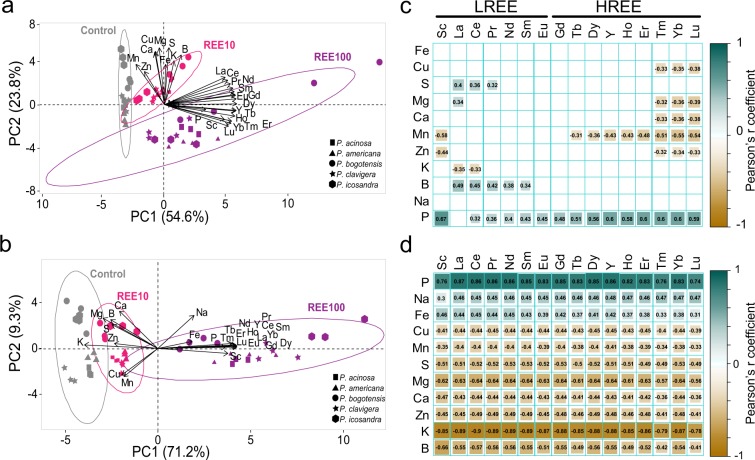
Principal component analysis and Pearson correlation matrices of REE, micro- and macro-elemental composition of leaves (**a**,**c**), and roots (**b**,**d**) of *Phytolacca* species. Plants were exposed to a mixture of 10 µM or 100 µM REEs (REE10 and REE100, respectively) or left unexposed (control). REE, macro- and micro-element concentrations were used as quantitative variables. For the Pearson correlation matrices, only significant correlations are shown (P < 0.05, BH adjustment).

Finally, the ionome of the different species under the three different exposure conditions was established (Supplementary Tables S1, S2). The two different compartments, leaves (Supplementary Tables S1a, S2a) and roots (Supplementary Tables S1b, S2b), were treated separately. Regarding the accumulation and fractionation of REEs, no differences were observed between the five species tested. Consequently, a global analysis was carried out using the ionomes of the different *Phytolacca* species to identify putative correlations between REE accumulation and essential element composition (Fig. 7). Two principal component analyses (PCA) (Fig. 7a,b) and correlation matrices were generated for both leaves and roots (Fig. 7c,d).

In the PCA for the leaves, the two first principal components accounted for 78.4% of the observed variations, with the first principal component accounting for 54.6% (Fig. 7a). The different conditions tested (control, REE10, and REE100) were well distinguished in the PCA. As expected, all the REEs were positively correlated together (Fig. 7a). Interestingly, in the second principal component analysis, we observed that LREEs and HREEs were grouped separately (Fig. 7a). This is in agreement with the HREE enrichment relative to the LREEs observed in the leaves (Fig. 4a). The first axis of the PCA was mostly structured by the REEs, contributing more than 82% each but also by P with 57% and Mn for 40%. In the second axis, S and Mg were the most structuring variables, with 91% of explained inertia, followed by Cu (85%), Ca (84%), B (80%), K (70%), Fe and Mn (65%). The segregation of REEs (first axis) from the other elements (second axis) on the PCA (Fig. 7a) is supported by the Pearson correlation matrix (Fig. 7c). Indeed, relatively few correlations were found between REE accumulation and the concentration of essential elements. Whereas no significant correlation was found between any of the REEs and Fe or Na, negative correlations were found between the heaviest REEs (Tm to Lu) and Cu, Ca, Mg, and Zn (Fig. 7c). A similar negative correlation was observed for Mn with HREEs (Gd excepted) (Fig. 7c). Conversely, positive correlations were found between P and all REEs except La. Similar positive correlations were also found between some LREEs and B and S (Fig. 7c). Interestingly, the Pearson’s r coefficient increased (P only) or decreased (the other elements), with the atomic number of REEs (Fig. 7c). Again, Sc did not follow the global rule observed for the other REEs, supporting its difference compared to the lanthanides and Y.

Correlations were more abundant and stronger between macro- or micronutrient elements and REEs in the root compartment (Fig. 7b–d). In the PCA, the two first principal components accounted for 80.5% of the observed variations, with the first principal component accounting for 71.2% (Fig. 7b). The first axis of the PCA was mostly structured by REEs, K, and P, contributing more than 84% each, and Mg contributed 68%. In the second axis, Ca was the most structuring variable, with 74% of explained inertia, followed by Na (65%), Mg (59%), B (56%), and S (47%). REEs covariated together and were also positively correlated with P in both roots and leaves. However, since (i) the nutritive solution lacked P during the exposure phase of REEs and (ii) growth at REE100 was reduced compared to the control, it was expected that P concentration in the biomass would be subjected to a dilution effect in the control treatment compared to the REE-exposed treatments. The observations reported in Supplementary Table S1 and Fig. 7c,d are in agreement with this hypothesis. While no correlation was found in the leaves between Fe or Na and REEs, positive correlations were observed in the roots (Fig. 7b–d). The other elements, namely, Cu, Mn, S, Mg, Ca, Zn, K, and B, were all negatively correlated with REEs. These correlations were highly influenced by the highest exposure condition (REE100) (Fig. 7b–d, Supplementary Tables S1, S2).

REEs have a similar ionic radius to that of Ca. This characteristic has resulted in the use of REEs as Ca-channel blockers^48,49^, and later on, it was suggested that La and Eu could enter into plant cells through Ca-channels^50^. Therefore, the negative correlation found between Ca and REEs is not unexpected. Such antagonistic effects between Ca and La have been previously reported in plants^41,51^. Yuan *et al*. (2017) also demonstrated that increasing concentrations of Ca inhibited REE accumulation in *P. americana*^21^. Along with Ca, Mg also had a negative correlation with REE contents. Such deprivation had already been reported for *P. americana* exposed to REEs^21^ but also in *Brassica napus* exposed to Ce^52^. Mg is necessary for chlorophyll synthesis and functioning; thus, the decrease in chlorophyll content could be related to the Mg reduction due to REE exposure. A negative correlation between REE and Mn was also found in leaves and roots (Fig. 7). *Phytolacca americana*^17–19^ and *P. acinosa*^25,26^ are two known Mn hyperaccumulators. It is interesting to note the reduced Mn content that occurred concomitantly with the accumulation of REEs. This might suggest a competitive uptake between these elements in *Phytolacca* species as in *P. acinosa* between Mn and Cd^53^.

## Conclusions

*Phytolacca* species are fast growing and high-biomass producing plants. *P. americana* is the first species of the genus that was demonstrated to be an REE accumulator. By testing five *Phytolacca* species under two different REE concentrations, this study therefore brings new information regarding the effect of REEs on the physiology and ionome of the REE-accumulating *Phytolacca* species. We used here hydroponic conditions to finely compare the potential of the different *Phytolacca* species to accumulate the whole set of REE species. Further research should be performed on the growth and REE accumulation potential of the different *Phytolacca* species with various REE-contaminated soils. These two parameters need to be evaluated in conditions of contamination to determine *in situ* the phytoextraction potential of the different *Phytolacca* species. It is interesting to note that the REE accumulating trait is conserved within the *Phytolacca* genus, as reported for the *Carya* genus^16^. Such information is very useful for the phytoremediation of REE-contaminated sites. Indeed, the different *Phytolacca* species tested here originate from different locations. For example, *P. bogotensis* is native to Colombia, *P. icosandra* to Mexico, and *P. acinosa* to Tibet^28^. Therefore, the conserved REE accumulation trait among these different species can be used to choose the most appropriate species according to the climatic and/or pedological context of the area to be remediated. Finally, to better understand the molecular and genetic mechanisms that underlie this particular trait, further work is needed.

## Materials and Methods

### Plant growth and REE treatments

Five *Phytolacca* species were used in the present study, including *P. acinosa* Roxb., *P. americana* L., *P. bogotensis* Kunth, *P. icosandra* L. and *P. clavigera* W.W. Smith. Seeds were obtained from Chiltern seeds (Wallingford, UK), the VanDusen Botanical Garden (Vancouver, Canada) and the Jean-Marie Pelt Botanical Garden (Villers-lès-Nancy, France). They were treated with 37% H_2_SO_4_ for 5 min and rinsed 5 times in sterile deionized water. Seeds were subsequently sown in Petri dishes on half-strength Murashige & Skoog agar medium. Four-week-old seedlings were transferred into a modified quarter-strength Hoagland nutrient solution (2 mM NH_4_NO_3_, 3 mM KNO_3_, 2 mM KCl, 2 mM Ca(NO_3_)_2_∙4H_2_O, 2 mM MgSO_4_∙7H_2_O, 1.5 mM NaCl, 600 µM KH_2_PO_4_, 100 µM CaCl_2_, 50 μM NaFe(III)-EDTA, 50 μM H_3_BO_3_, 5 μM MnCl_2_∙4H_2_O, 10 µM ZnSO_4_∙7H_2_O, 0.5 μM CuSO_4_∙5H_2_O, 0.1 μM Na_2_MoO_3_, pH 5.6)^54^. Two-litre black containers were filled with nutritive solution that was continuously aerated. Six-week-old seedlings were subsequently transferred to the same nutritive solution containing REEs or not (control medium) and lacking KH_2_PO_4_ to avoid REE precipitation with phosphates^55^. Promethium was omitted since it is only available under its radioactive form. Two different total concentrations (10 and 100 µM) of a mixture of 16 REEs were used. All REEs were therefore applied at equimolar concentrations (625 nM or 6.25 µM). REE tri-chloride salts were used (Sc, Y, La, Ce, Pr, Nd, Sm, Eu, Gd, Tb, Dy, Ho, Er, Tm, Yb and Lu) and were purchased from Sigma-Aldrich (Saint-Quentin-Fallavier, France). Three or four plants (control and 10 µM REE treatments, n = 3; 100 µM REE treatments, n = 4) per species and per treatment were grown individually in hydroponic containers in a growth chamber under controlled conditions: 18/23 °C, 12/12 h dark/light cycles (light intensity 260 µmol photons/m^2^/s), respectively.

After 21 days of exposure, plants were harvested. Roots were first rinsed in water, followed by a 15 min incubation in 5 mM ice-cold CaCl_2_, washed twice in deionized water and finally blotted dry carefully with tissue paper. Root architecture and leaf pigments were analysed (see below). Subsequently, plants were separated into roots, stems and leaves and weighed. Finally, the samples were ground into thin powder using liquid nitrogen and stored at −80 °C until use. Aliquots of powder samples were also dried at 70 °C for 2 days, weighed and further used for elemental analysis (see below).

The solutions were analyzed (see below) at the beginning and at the end of the experiment. The nominal and measured concentrations were very similar at the beginning of the experiment. Indeed, when compared to the nominal concentrations of 10 or 100 µM, the measured total REE concentrations varied only by a mean of 5.3 and 5.5%, respectively. At the end of the experiment, the REE concentrations were similar to the initial concentrations. As an example, after three weeks of incubation, the concentrations varied from 6.5% (100 µM treatment) to 11.4% (10 µM treatment) from the initial concentrations (data not shown). Even though plants took up a fraction of the REEs, this part did not contribute to deplete the medium and was counterweighed by a slight reduction of the volume of the growth medium due to evapotranspiration. As a matter of fact, the plants were exposed to similar concentrations all over the incubation period.

### Species identification

Cellular extracts from the different *Phytolacca* species were obtained using the REDextract-N-Amp™ Plant PCR kit (Sigma-Aldrich) according to the manufacturer’s protocol. The internal transcribed spacer (ITS) region was amplified using the primers ITS1 and ITS4^56^. Twenty microliters of a mixture containing 0.2 µM of each primer, 4 µl of plant extract, 5.6 µl of H_2_O and 10 µl of REDExtract-N-Amp PCR ready mix were used for PCR amplification. The following PCR programme was used: 3 min at 94 °C, followed by 40 cycles of 94 °C for 1 min, 55 °C for 1 min and 72 °C for 1 min, and 10 min at 72 °C. PCR products were cloned into the pGEM-T® Easy vector system I (Promega, Charbonnières-les-Bains, France), and the corresponding plasmids were sequenced. Related *Phytolacca* sequences were retrieved from the GenBank database using the NCBI BLAST program, and ClustalW was used to align sequences. The maximum likelihood tree was drawn with the kimura 2-parameter model using MEGA 7^57^. Bootstrap analysis was performed with 1,000 replications.

### Analysis of biomarkers

A series of biomarkers were used to assess the potential toxic effects of REEs on *Phytolacca* species. First, leaf pigments (chlorophylls, flavonoids and anthocyanins) and the nitrogen balance index (NBI) were quantified using a DUALEX^®^ SCIENTIFIC^+^ apparatus^58^ (Force-A, Orsay, France) before harvesting. Forty technical replicates were carried out per plant. Second, root architecture was analysed. Root systems were photographed at harvest. The analyses of lateral root length and lateral root density (number of lateral roots per cm of primary root) were carried out using the RootNav software^59^. Finally, the leaf and root contents of malondialdehyde (MDA) and total antioxidant compounds (TAC) were assayed from 50 mg of frozen powder using commercial kits (MAK085 and MAK187, Sigma-Aldrich, France) according to the manufacturer’s protocols. Two technical replicates were performed per sample.

### Elemental analysis

The quantification of elements was performed using 250 mg of dried plant powder. Mineralization was carried out using 3.5 mL nitric acid (HNO_3_, 65%, analytical grade, Optima) and 1 mL hydrogen peroxide, and samples were incubated in a heating block digestion system (DigiPREP, SCP Sciences, Courtaboeuf, France). A gradual heating mode was used to achieve a final temperature of 100 °C (total run of 265 min). Then, ultrapure water (18.2 MΩ/cm^2^, Millipore Milli-Q Integral 3 system, Molsheim, France) was added to a final volume of 25 mL, and samples were filtrated to 1 μm. Elemental concentrations were determined by ICP-AES (Radial ICAP 6500 Model, Thermo Fischer Scientific, Courtaboeuf, France) or ICP-MS (X Series II Model, Thermo Fischer Scientific, Courtaboeuf, France) for REEs. Oriental basma tobacco leaves (INCT-OBTL-5, LGC Promochem, Molsheim, France) were used as certified reference material.

### Statistical analyses

One-way ANOVA and Tukey’s HSD post hoc test were used when normality of data (Shapiro-Wilks test) and homoscedasticity (Levene’s test) conditions were respected. When at least one condition was rejected, data were subsequently analysed using one-way non-parametric Kruskal-Wallis test followed by Wilcoxon post hoc test. The rejection level was set at α = 0.05 in all analyses. Percentage values were *arcsin*-transformed before statistical analyses. All analyses were performed using R software (version 3.4.1).

## Supplementary information

tables S1 and Table S2
