# Supplementary material for: Accumulation and fractionation of rare earth elements are conserved traits in the Phytolacca genus
Source: Sci Rep. 2019 Dec 5;9:18458. doi: 10.1038/s41598-019-54238-3 (PMC6895054; doi:10.1038/s41598-019-54238-3)
Supplement: Supplementary file 1 — tables S1 and Table S2 [file 41598_2019_54238_MOESM1_ESM.docx]

**Supplementary information:**

Title: **Accumulation and fractionation of rare earth elements are conserved traits in the *Phytolacca* genus**

Authors: Nicolas Grosjean, Marie Le Jean, Charlotte Berthelot, Michel Chalot, Elisabeth Maria Gross, Damien Blaudez

**Supplementary Table S1. Macroelement composition (mg/g DW) of leaves (a) and roots (b) of *Phytolacca* species exposed to REEs.** *Phytolacca* species were exposed to a mixture of 10 µM or 100 µM REEs (REE10 and REE100, respectively). Values are means ± SD (n=3 or n=4). Within a given species, significant differences between treatments are indicated by different letters (P<0.05, ANOVA Tukey’s HSD).

**Supplementary Table S2. Microelement composition (µg/g DW) of leaves (a) and roots (b) of *Phytolacca* species exposed to REEs.** *Phytolacca* species were exposed to a mixture of 10 µM or 100 µM REEs (REE10 and REE100, respectively). Values are means ± SD (n=3 or n=4). Within a given species, significant differences between treatments are indicated by different letters (P<0.05, ANOVA Tukey’s HSD).
